# A study on prevalence of microbial contamination on the surface of raw salad vegetables

**DOI:** 10.1007/s13205-016-0585-5

**Published:** 2017-04-08

**Authors:** Sujeet Kumar Mritunjay, Vipin Kumar

**Affiliations:** Applied Microbiology Laboratory, Department of Environmental Science and Engineering, Indian Institute of Technology (Indian School of Mines), Dhanbad, Jharkhand 826 004 India

**Keywords:** Raw salad vegetables, *E. coli* O157:H7, *L. monocytogenes*, *Salmonella* sp., *Exiguobacterium* sp.

## Abstract

The present work evaluates the microbiological quality of raw salad vegetables (RSV) consumed in Dhanbad city, India. A total of 480 samples of 8 different raw salad vegetables from local market were examined for overall microbial quality in terms of aerobic mesophilic, psychrotrophic counts, yeast, mould and total coliform levels. *E. coli* O157:H7, *Listeria monocytogenes* and *Salmonella* sp. were detected by real-time polymerase chain reaction (qPCR) subsequent to isolation. Results showed that all the samples were found positive for total coliform; however, *E. coli* was detected in 16.7% of the total samples. Pathogenic microorganisms such as *E. coli* O157:H7, *L. monocytogenes* and *Salmonella* spp. were detected in 1.3, 3.5 and 4.0%, respectively, of the total samples. However, pathogens were not detected in any of the cabbage samples. The *Exiguobacterium* sp. (Strain ISM SP 2014) was detected in the spinach sample while studying the bacterial contamination, reported for the first time on the surface of RSV. The 16S rRNA gene sequencing showed less than 92% similarity with sequences available in the public domain.

## Introduction

Raw salad vegetables (RSV) are an essential ingredient of a healthy diet, and the demand for salad vegetables has increased in recent years. Nutritionists emphasize the importance of raw vegetables in healthy diets, and researchers and governmental publicity campaigns around the world tend to recommend consumption of at least five servings of fruits and vegetables per day. In contrast to their health benefits, the consumption of fresh vegetables has also been associated with risk for consumers (Weldezgina and Muleta 2016). Vegetables are rich in carbohydrates, anti-oxidants, minerals, vitamins and fibres (Said 2012) and often consumed uncooked. The phytonutrients can act as effective media for the transmission of pathogens (Khan et al. 1992; Abougrain et al. 2010). RSV can become contaminated with pathogenic micro-organisms whilst growing in fields, or during harvesting, post-harvest handling, processing and distribution. Different agronomic practices can contaminate vegetables in various stages. Contamination occurs mostly before harvesting, either by contaminated manure, sewage, irrigation water, and wastewater from livestock operations or directly from wild and domestic animals or during harvesting, transport, processing, distribution, and marketing or even at home (Eraky et al. 2014; Rahman et al. 2014; Pagadala et al. 2015; Maffei et al. 2016). The use of wastewater for irrigation affects the quality of RSV and human health. It could be the possible source of pathogenic microorganisms on vegetables. Pathogens like *Salmonella* sp., *Shigella* sp., *Campylobacter* sp., *L. monocytogenes* and *E. coli* O157:H7 can contaminate RSV through contact with sewage and manure. Contaminations may also occur after harvest through dirty wash water, by cross-contamination from an infected food-handler (Sivapalasingam et al. 2004), and their consumption either raw or uncooked can be the important risk factor for the transmission of pathogens (Said 2012). Microorganisms that adhere to the surface of the vegetables are mainly Gram-negative saprophytes that may survive even after washing and sanitizing steps due to the formation of biofilms on the surface of the vegetable or from protection by the cuticle of the vegetable (Seo et al. 2010).

The human infections associated with consumption of raw fruits and vegetables have increasedduring the past decade (Eraky et al. 2014) and have been recognized as a means of transmission of foodborne pathogens (Pagadala et al. 2015). The factors contributing to this increase may include changes in agronomic practices and an increase in the number of immune-compromised consumers (Eraky et al. 2014). Many reports specified that pathogenic bacteria may adhere to the surface of raw vegetables and cause foodborne outbreaks or chronic infections (Oliveira et al. 2011). Foodborne illnesses caused by *E. coli* are one of the most important gastrointestinal diseases and represent a public health risk. Its presence on the surface of RSV indicates the contamination of foecal origin. Therefore, the presence of *E. coli* can be used to evaluate the microbiological quality of raw vegetables.

A number of national microbiological guidelines have been published in many countries such as the UK, France, Japan, Korea (Seo et al. 2010) and Singapore (AVA 2005). In India, Food Safety and Standards Authority of India (FSSAI 2006) have published a regulation which covers food safety in the country.

There has been an increasing number of fresh produce-associated foodborne illnesses identified internationally and efforts are being made to resolve these food safety problems (Denis et al. 2016). However, information on the microbiological quality of RSV, especially with respect to incidence of bacterial pathogens, are not well documented in the city of Dhanbad, India. This study aimed to evaluate the microbial contamination on the surface of unwashed RSV consumed in Dhanbad city of India.

## Materials and methods

### Study area

Dhanbad city (23.8°N 86.45°E), known as the coal capital of India, is situated in the state of Jharkhand, India. The district is highly populated and rich in terms of coal-based industries. Historically, it was the most productive agricultural tract of the country and its agriculture maintained a lead over other regions. But presently, agricultural development in this region is much below its potential. Agriculture in the district is mostly rain fed and comprises a monocrop area. The average annual rainfall in the district is approximately 1300 mm. The city is producing as well as importing the salad vegetables from nearby villages and cities.

### Sample collection

A total of 480 samples of raw salad vegetables (*n* = 60, each of cucumber, tomato, carrot, coriander, cabbage, beet-root, radish, and spinach) were collected during July 2013 to June 2014 from the retail market. Samples were collected on different days and different vendors, some of them from local producers and others from different retailers. The samples were transported promptly to the laboratory in ice boxes and analysed within 24 h following collection. Damaged samples were discarded. Each vegetable sample was placed in a separate sterile nylon bag and labelled with a unique number and date of collection thus making the results of this study more specific.

### Evaluation of the samples

To suspend the phyllosphere-associated microorganisms from the surface of cucumber, tomato, spinach coriander and cabbage, aliquots of 225 mL sterile 0.1% buffered peptone water (BPW) were added to each nylon bag containing vegetable sample (25 g). For other samples, i.e. carrot, beet-root and radish, each sample was aseptically transferred into a nylon bag and filled with the equal weight of BPW. Samples were then agitated and rubbed separately in the nylon bag for 2 min to suspend surface microbes (Seow et al. 2012).

### Microbiological analysis

Aerobic mesophilic, psychrotrophic microorganisms, total coliforms, yeast and mould were determined using the standard protocol. Four tenfold serial decimal dilutions were made for each sample; 1 mL of each step was inoculated into duplicates of plate count agar (PCA) (Himedia) (Sospedra et al. 2013). For mesophilic plate count, the plates were incubated at 37 °C for 24 h, and for the psychotropic plate count, incubated at 6 °C for 5–7 days (Seow et al. 2012). The colonies formed on the plates were counted and expressed as log_10_ colony-forming unit/g (log cfu/g). To enumerate the *E. coli*, 1 mL of diluted sample was spread-plated onto eosin methylene blue (EMB) agar (Himedia, India) and plates were incubated at 37 °C for 24 h. Estimation of yeast and mould was performed using Dichloran Rose Bengal Chloramphenicol (DRBC) Agar (Himedia) containing 0.1% chloramphenicol by the spread plate technique at 25 °C for 5 days, following which the numbers of colonies were counted in terms of log cfu/g of the samples.

### Detection of *E. coli* O157:H7, *Salmonella* sp. and *Listeria monocytogenes*

*E. coli* O157:H7 was detected using the reference method described in International Organization for Standardization (ISO 16654:2001). In brief, 25 g of each sample was diluted in 225 mL of modified tryptone soya broth (TSB; Oxoid) added with novobiocin, homogenized for 2 min and incubated for 24 h at 41.5 °C. After enrichment, the selective and differential isolation of *E. coli* O157:H7 was streaked onto Tellurite cefixime sorbitol macconkey agar (TC-SMAC) (Oxoid).

Detection of *Salmonella* sp. was carried out as per ISO 6579:2000 reference method. In brief, 1 and 0.1 mL of homogenate BPW were inoculated into tetrathionate (TT) broth with Novobiocin (Himedia) and Rappaport–Vassiliadis (RV) broth (Himedia), respectively. The enriched broth was incubated for 24 ± 2 h, respectively, at 37 ± 1 °C (for TT broth) and 42 ± 1 °C (for RV broth). The positive cultures were streaked onto XLD Salmonella agar (Himedia) at 37 ± 1 °C for 24 h and confirmed using the API 20E kit (Biomerieux) (Seow et al. 2012; Sospedra et al. 2013).

*L. monocytogenes* was detected as per ISO 11290-1 (1996). 25-g samples were weighed into sterile nylon bags and homogenized with 225 mL of Fraser broth (Himedia). After homogenizing and pre-culturing at 37 ± 1 °C for 48 ± 2 h, the positive broth was streaked onto Listeria Palcam agar (Himedia) and incubated at 37 ± 1 °C for 24 ± 2 h. Characteristic colonies were Gram stained and tested for motility, oxidase and catalase activity followed by identification with the API Listeria system (Biomerieux, Marcy I’Etoile, France).

### Detection of pathogens by qPCR

Detection of *Salmonella* sp., *L. monocytogenes* and *E. coli* O157:H7 was confirmed by real-time quantitative PCR (qPCR) after selective enrichment steps. The analyses were performed as per ISO 7579 (1997, revised 2002). Samples were homogenized in BPW, incubated at 37 °C for 24 h (pre-enrichment) and subsequently enriched broth media, as described in the above section. After these enrichment steps, 100-µL aliquots of the samples were diluted 1:10 (v/v) in sterile distilled water and kept at −20 °C for qPCR, while 500-µL aliquots were mixed with 500 µL of 400 g L^−1^ sterile glycerol and kept at −20 °C for pathogen detection from qPCR positive samples. For all tested raw vegetables, the detection limit was the same as using the standard ISO protocol. A method targeting the invasion invA gene [primers Strinva-JHO-2F/R and Strinva-JHO-2p TaqMan probe (FAM and TAMRA dual-labelled)], previously reported by Hoorfar et al. (2000), was used to detect pathogens. Uniplex reactions were performed using a PCR Core Reagents Kit (Applied Biosystems–Roche Molecular Systems Inc., Branchburg, Germany). Reactions were run on an ABI PRISM 7900HT Sequence Detection System. All samples were analysed in triplicate. Both the negative (distilled water + all PCR components) and positive (a DNA template + all PCR components) controls were included in each run.

### Detection of *Exiguobacterium* sp. and 16S rDNA gene sequence

Decimal dilution of the sample was spread plated onto nutrient media (Himedia, India) and plates were incubated at 37 °C for 48 h, as this media was reported to be efficient for mass production of *Exiguobacterium* sp. (Manon et al. 2015). The DNA isolation was carried out according to the standard protocol (Morales and Holben 2009; Fulzele et al. 2011). The PCR assay was performed using 704F and 907R universal primer by Xcelris Labs (India). The rDNA contigs (16s) assay using NCBI-BLAST to find the closest match of the contigs sequence. Phylogenetic tree was constructed using MEGA7 software using the neighbour joining method. Strain ISM SP 2014 possessed most of the phenotypic properties of the genus *Exiguobacterium* and, based on 16S rRNA gene sequence analysis, was assigned to this genus.

### Quality control and quality assurance

Analytical grade chemicals and culture media, double-distilled, deionized water and Milli-Q Millipore water were used for preparation of all reagents and calibration standards. Calibrated glassware was used for experimental work. To avoid other microbial contamination, special care was taken to transfer the samples from the sampling site to the laboratory.

### Legislation

The microbial quality of the food was evaluated based on the specifications of the Indian Food Safety and Standards Act, 2006 (FSSAI 2006). According the Act, microorganism of concern is not permitted in the 25 g of food.

### Statistical analysis

The number of colonies per plate was converted to colony-forming unit (cfu/g) and log-transformed (log_10_) to obtain normal distributions for statistical analysis. The data were analysed by descriptive statistics procedures of SPSS Version 16.0 (Statistical Package for the Social Sciences (SPSS Inc.), Chicago, USA) and by using Origin 8.0 software (OriginLab Corporation, Northampton, MA, USA) to plot box and whisker diagrams for each microbiological count.

## Results and discussion

**Fig. 1:**
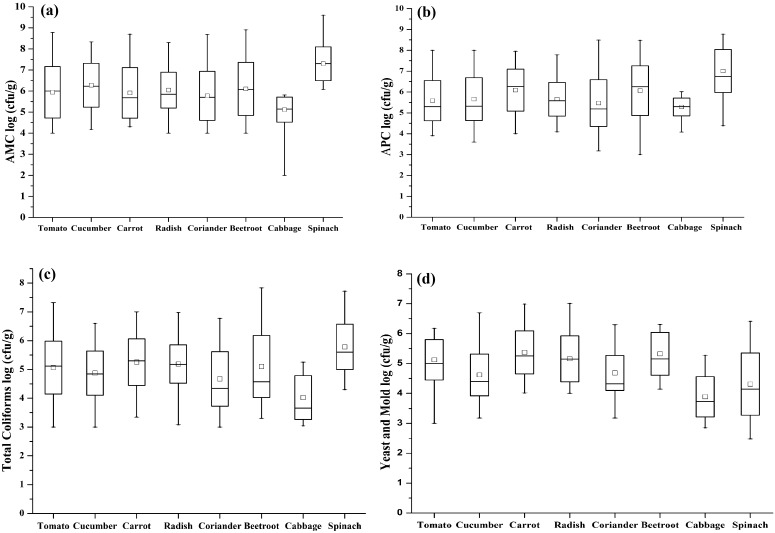
Box and whisker diagrams of **a** aerobic mesophilic count, **b** aerobic psychrotrophic count, **c** total coliforms count and **d** yeast and mould count. *Horizontal bars* indicate mean values, *boxes* show the standard deviation, *whisker lines* indicate the ranges and *box* shows median values

Results of microbial contamination of unwashed RSV are documented in Tables 1, 2, 3 and 4 and Fig. 1. The results showed that there was a significant variation between samples of each RSV. The aerobic mesophilic plate count of RSV samples was presented in Table 1. The mean AMC for all tested samples was 6.1 log cfu/g, ranging from 2.0 to 9.6 log cfu/g. Most of the tested samples were found with AMC in the range of 6.0–8.0 log cfu/g. The results reflect that 85.4% (*n* = 480) of the samples exceeded the limits for aerobic bacterial count, indicating the samples were unacceptable for consumption, since the aerobic bacterial count for food should not be higher than 5.0 log cfu/g in India and several other countries (European Union 2005, 2007; FSSAI 2006). The samples with the highest microbial counts were spinach and cucumber, while cabbage had the the lowest. All cabbage samples were observed to have less than 6.0 log cfu/g with a range of 2.0–5.8 log cfu/g and 30% samples demonstrated to have less than 5.0 log cfu/g, whereas spinach was most contaminated among all observed commodities with highest mean count of 7.3 log cfu/g with a frequency of 6.1–9.6 log cfu/g. 282 (58.8%) samples of RSV, excluding cabbage, fell between 5.0 and 8.0 log cfu/g. The high mean count may indicate the poor handling practice during storage and point of selling. Several studies reported that the major source of microbial contamination was an anthropogenic disturbance in human, animals and irrigation water (Oliveira et al. 2011). The aerobic mesophilic mean count for radish was observed 6.0 log cfu/g along with cucumber and beet-root. This corroborates previous the results of study conducted by Pingulkar et al. (2001), where the mean counts of radish and cucumber were 1.0 × 10^6^ (6.0 log cfu/g) and 1.7 × 10^6^ (6.2 log cfu/g), respectively, similar to the present study. Our study also showed a similar trend in the case of raw salad vegetables with those reported for fresh cut organic vegetables and ready-to-eat salad (i.e. aerobic plate counts lower than 8.3 log cfu/g) (Nguz et al. 2005; Pingulkar et al. 2001).

**Table 1:** Population of the aerobic mesophilic microorganisms

Sample	*n*	<10^5a^	10^5^–10^6^	10^6^–10^7^	10^7^–10^8^	>10^8^	Range^a^	Mean^a^ ± SD
Tomato	60	11 (18.3%)	17 (28.3%)	22 (36.7%)	05 (8.3%)	05 (8.3%)	4.0–8.8	5.9 ± 1.2
Cucumber	60	07 (11.7%)	12 (20.0%)	25 (41.7%)	11 (18.3%)	05 (8.3%)	4.3–8.7	6.3 ± 1.1
Carrot	60	15 (25.0%)	16 (26.7%)	18 (30.0%)	06 (10.0%)	05 (8.3%)	4.3–8.7	5.9 ± 1.2
Radish	60	04 (6.7%)	26 (43.3%)	17 (28.3%)	12 (20.0%)	01 (1.7%)	4.0–8.3	6.0 ± 0.9
Coriander	60	08 (13.3%)	29 (48.3%)	14 (23.3%)	04 (6.7%)	05 (8.3%)	4.0–8.7	5.8 ± 1.2
Beet-root	60	07 (11.7%)	22 (36.7%)	16 (26.7%)	11 (18.3%)	04 (6.7%)	4.0–8.9	6.1 ± 1.3
Cabbage	60	18 (30.0%)	42 (70.0%)	00 (0.0%)	00 (0.0%)	00 (0.0%)	2.0–5.8	5.1 ± 0.6
Spinach	60	00 (0.0%)	00 (0.0%)	23 (38.3%)	27 (45.0%)	10 (16.7%)	6.1–9.6	7.3 ± 0.8
Total	480	70 (14.6%)	164 (34.2%)	135 (28.1%)	76 (15.8%)	35 (7.3%)	2.0–9.6	6.1 ± 1.2

*n* number of samples, *SD* standard deviation

^a^The unit of number is log cfu/g

**Table 2:** Population of the aerobic psychrotrophic microorganisms

Sample	*n*	<10^5a^	10^5^–10^6^	10^6^–10^7^	10^7^–10^8^	>10^8^	Range^a^	Mean^a^ ± SD
Tomato	60	10 (16.7%)	38 (63.3%)	02 (3.3%)	09 (15.0%)	01 (1.7%)	3.9–8.0	5.6 ± 1.0
Cucumber	60	14 (23.3%)	22 (36.7%)	15 (25.0%)	08 (13.3%)	01 (1.7%)	3.6–8.0	5.7 ± 1.0
Carrot	60	11 (18.3%)	10 (16.7%)	28 (46.7%)	11 (18.3%)	00 (0.0%)	4.0–8.0	6.1 ± 1.0
Radish	60	12 (20.0%)	23 (38.3%)	21 (35.0%)	04 (6.7%)	00 (0.0%)	4.1–7.8	5.7 ± 0.8
Coriander	60	19 (31.7%)	22 (36.7%)	11 (18.3%)	06 (10.0%)	02 (3.3%)	3.2–8.5	5.5 ± 1.1
Beet-root	60	10 (16.7%)	16 (26.7%)	20 (33.3%)	12 (20.0%)	02 (3.3%)	3.0–8.5	6.1 ± 1.2
Cabbage	60	05 (8.3%)	52 (86.7%)	03 (5.0%)	00 (0.0%)	00 (0.0%)	4.1–6.0	5.3 ± 0.4
Spinach	60	03 (5.0%)	04 (6.7%)	25 (41.7%)	18 (30.0%)	10 (16.7%)	4.4–8.8	7.0 ± 1.0
Total	480	84 (17.5%)	187 (39.0%)	125 (26.0%)	68 (14.2%)	16 (3.3%)	3.0–8.5	5.9 ± 1.0

*n* number of samples, *SD* standard deviation

^a^The unit of number is log cfu/g

**Table 3:** Population of the total coliforms

Sample	*n*	<10^4a^	10^4^–10^5^	10^5^–10^6^	10^6^–10^7^	<10^7^	Range^a^	Mean^a^ ± SD
Tomato	60	05 (8.3%)	21 (35.0%)	23 (38.3%)	10 (16.7%)	01 (1.7%)	3.0–7.3	5.1 ± 0.9
Cucumber	60	01 (1.7%)	31 (51.7%)	18 (30.0%)	10 (16.7%)	00 (0.0%)	3.0–6.6	4.9 ± 0.8
Carrot	60	01 (1.7%)	20 (33.3%)	25 (41.7%)	13 (21.7%)	01 (1.7%)	3.3–7.0	5.3 ± 0.8
Radish	60	01 (1.7%)	14 (23.3%)	39 (65.0%)	06 (10.0%)	00 (0.0%)	3.1–7.0	5.2 ± 0.7
Coriander	60	07 (11.7%)	36 (60.0%)	08 (13.3%)	09 (15.0%)	00 (0.0%)	3.0–6.8	4.7 ± 0.9
Beet-root	60	01 (1.7%)	30 (50.0%)	11 (18.3%)	14 (23.3%)	04 (6.7%)	3.3–7.8	5.1 ± 1.1
Cabbage	60	35 (58.3%)	11 (18.3%)	14 (23.3%)	00 (0.0%)	00 (0.0%)	3.0–5.3	4.0 ± 0.8
Spinach	60	00 (0.0%)	07 (11.7%)	33 (55.0%)	18 (30.0%)	02 (3.3%)	4.3–7.7	5.8 ± 0.8
Total	480	51 (10.6%)	170 (35.4%)	171 (35.6%)	80 (16.7%)	08 (1.7%)	3.0–7.8	5.0 ± 0.9

*n* number of samples, *SD* standard deviation

^a^The unit of number is log_10_ cfu/g

**Table 4:** Population of the yeast and mould

Sample	*n*	<10^4a^	10^4^–10^5^	10^5^–10^6^	10^6^–10^7^	>10^7^	Range^a^	Mean^a^ ± SD
Tomato	60	01 (1.7%)	17 (28.3%)	39 (65.0%)	03 (5.0%)	00 (0.0%)	3.0–6.2	5.1 ± 0.7
Cucumber	60	03 (5.0%)	45 (75.0%)	10 (16.7%)	02 (3.3%)	00 (0.0%)	3.2–6.7	4.6 ± 0.7
Carrot	60	00 (0.0%)	22 (36.7%)	21 (35.0%)	17 (28.3%)	00 (0.0%)	4.0–7.0	5.4 ± 0.7
Radish	60	00 (0.0%)	24 (40.0%)	21 (35.0%)	13 (21.7%)	02 (3.3%)	4.0–7.0	5.2 ± 0.8
Coriander	60	01 (1.7%)	29 (48.3%)	29 (48.3%)	01 (1.7%)	00 (0.0%)	3.2–6.3	4.7 ± 0.6
Beet-root	60	00 (0.0%)	22 (36.7%)	18 (30.0%)	20 (33.3%)	00 (0.0%)	4.1–6.3	5.3 ± 0.7
Cabbage	60	32 (53.3%)	26 (43.3%)	02 (3.3%)	00 (0.0%)	00 (0.0%)	2.8–5.3	3.9 ± 0.7
Spinach	60	20 (33.3%)	24 (40.0%)	10 (16.7%)	06 (10.0%)	00 (0.0%)	2.5–6.4	4.3 ± 1.0
Total	480	57 (11.9%)	209 (43.5%)	150 (31.3%)	62 (12.9%)	02 (0.4%)	2.8–7.0	4.8 ± 0.8

*n* number of samples, *SD* standard deviation

^a^The unit of number is log cfu/g

In this study, the trends of psychrotrophic counts were very similar to mesophilic counts (Table 2), with the highest mean count of spinach (7.0 log cfu/g) and lowest on cabbage (5.3 log cfu/g). The population of aerobic mesophilic microorganisms present was closely similar to that of aerobic psychrotrophic microorganisms and as a result, most of the microorganisms were able to grow in storage temperature. Garg et al. (1990) reported that storage at refrigeration temperatures generally promotes the growth of psychrotrophic microorganisms. However, mesophilic microorganisms may continue to grow at low temperatures, albeit at reduced growth rates. Such a trend was observed in this present study, where psychrotrophic counts are almost similar to mesophilic counts. This is in contrast to a study conducted by Seow et al. (2012), where psychrotrophic counts were reported to be considerably lower than mesophilic counts with highest mean count of 4.9 log cfu/g.

In this study, coliforms were detected in all the samples. RSV had mean counts of coliform ranging from 3.0 to 7.8 log cfu/g (Table 3). Among the leafy vegetables (spinach, cabbage and coriander), spinach has high mean count, 5.8 ± 0.8, whereas coriander and cabbage have mean count of 4.7 ± 0.9 cfu/g and 4.0 ± 0.8 log cfu/g, respectively. Between the root vegetables (beet-root, radish, carrot), carrot has a higher mean count of 5.3 ± 0.8 log cfu/g followed by radish (5.2 ± 0.7 log cfu/g) and beet-root (5.1 ± 1.1 log cfu/g), while tomato and cucumber have a mean count of 5.1 ± 0. 9 and 4.9 ± 0.8 log cfu/g, respectively. The possible explanation for leafy vegetables is that cabbage has densely leaved heads and spinach and coriander have open leaves which touch the soil regularly in the field with manure or irrigation water. Spinach leaves have large surface area, making them more susceptible to bacterial contaminations and adhesions, whereas coriander has small leaves, and its open leaves may also be in direct contact with soil and irrigation. These results are similar to those of many investigations where most of the salad vegetables showed coliform counts from 4 to 6 log cfu/g (Badosa et al. 2008; Seo et al. 2010; Seow et al. 2012). Similar to the present study, Seo et al. (2010) reported that 68% of the analysed samples had more than 4.0 log cfu/g with most samples having counts of 6.0–7.9 log cfu/g. Local sanitary conditions during production, post-production, transportation, storage and retail of fresh vegetables could also have contributed to the differences. Total coliform counts are considered as hygiene indicator, especially for foecal contamination. However, their presence does not indicatethe presence of pathogenic microorganism (Nguz et al. 2005). The observed mean counts of yeast and mould were lower than that of aerobic bacteria (Table 4). A study conducted by Seow et al. (2012) showed that the levels of bacteria isolated in tomato were lower than that of yeast and mould. In the present study, yeast and mould counts ranged from 0.3 to 5.5 log cfu/g with most of the samples having counts of 1–3 log cfu/g, in contrast to the study carried out by Badosa et al. (2008) that reported yeast and mould counts for fruits as ranging from <1 to 8 log cfu/g, with most samples having counts of 3–5 log cfu/g. Abadias et al. (2008) and Tournas (2005) obtained similar results with samples of fresh and minimally processed vegetables and sprouts. However, there are possible health problems associated with the presence of mould found in vegetables, as some mould may produce mycotoxins or induce allergies (Tournas 2005). RSV had signed (*p* < 0.05) higher aerobic mesophilic count and total coliform count (Table 5), this is in agreement with previous data (Badosa et al. 2008; Seow et al. 2012). Box and whisker plots (Fig. 1a–d) showed that mean aerobic mesophilic count values ranged from 2.0 to 9.6 and from 3.0 to 7.8 log cfu/g for total coliforms.

**Table 5:** Mean comparisons between different microbiological analyses

Sample	*n*	AMC^a^	APC^a^	Total coliforms^a^	Yeast and mould count^a^
Tomato	60	5.9^A^	5.6^B^	5.1^C^	5.1^C^
Cucumber	60	6.3^A^	5.7^B^	4.9^C^	4.6^C^
Carrot	60	5.9^A^	6.1^A^	5.3^B^	5.4^B^
Radish	60	6.0^A^	5.7^B^	5.2^C^	5.2^C^
Coriander	60	5.8^A^	5.5^A^	4.7^B^	4.7^B^
Beet-root	60	6.1^A^	6.1^A^	5.1^B^	5.3^B^
Cabbage	60	5.1^A^	5.3^A^	4.0^B^	3.9^B^
Spinach	60	7.3^A^	7.0^A^	5.8^B^	4.3^C^

*n* Number of samples

Means having different letters (A–C) are significantly different (*p* < 0.05)

^a^The unit of number is log cfu/g

In this study, *E. coli* population was noticed in 16.7% of total samples analysed. Populations of *E. coli*, often measured for monitoring the sanitary condition of food and an indicator of contamination of fecal origin (Santosa et al. 2012). However, *E. coli* was not detected in cabbage, but it was present in 9 samples (15%) of tomato, 9 (15%) of cucumber, 12 (20%) of carrot, 12 (20%) radish, 3 (5%) coriander, 11 (18.3%) of beet-root, 3 (5%) and 21 (35%) of spinach (Table 6). The incidence of *E. coli* in spinach was higher than in other RSV. In contrast to the present study, Pagadala et al. (2015) found that 5.4% (*n* = 259) of tomato was positive for generic *E. coli* in Maryland, Delware and New Jersey of United States. In the city of Pachuca, Hidalgo state of Mexico, Gomez-Aldapa et al. (2016) reported that 47% (*n* = 100) of coriander samples were contaminated with generic *E. coli* and the concentrations ranged between <3 and 1100 MPN/g. In Aizawl city of India, Chellapandi et al. (2015) observed that 4.88% (*n* = 27) of fresh vegetables (tomato, potato and cabbage) contained *E. coli*. The most possible causes of presence of *E. coli* on the surface of salad vegetables were as follows: sprinkling of contaminated water, the so-called grey water and growing the vegetables on contaminated soil, with contaminated irrigation water (Singh et al. 2006; Tyrrel et al. 2006; Pagadala et al. 2015).

**Table 6:** Detection of *E. coli*, *E. coli* O157:H7, *L. monocytogenes, Salmonella* sp. and *Exiguobacterium* sp. (ISM SP 2014)

Sample	*n*	*E. coli*	*E. coli* O157:H7	*L. monocytogenes*	*Salmonella* sp.	*Exiguobacterium* sp.
Tomato	60	09 (15.0%)	ND	04 (6.7%)	03 (5.0%)	ND
Cucumber	60	09 (15.0%)	ND	03 (5.0%)	03 (5.0%)	ND
Carrot	60	12 (20.0%)	ND	01 (1.7%)	02 (3.3%)	ND
Radish	60	12 (20.0%)	ND	01 (1.7%)	01 (1.7%)	ND
Coriander	60	03 (5.0%)	ND	ND	01 (1.7%)	ND
Beet-root	60	11 (18.3%)	02 (3.3%)	ND	02 (3.3%)	ND
Cabbage	60	03 (5.0%)	ND	ND	ND	ND
Spinach	60	21 (35.0%)	04 (6.7%)	08 (13.3%)	07 (11.7%)	02 (3.3)
Total	480	80 (16.7%)	06 (1.3%)	17 (3.5%)	19 (4.0%)	02 (0.4)

*n* number of samples; *ND* not detected

**Fig. 2:**
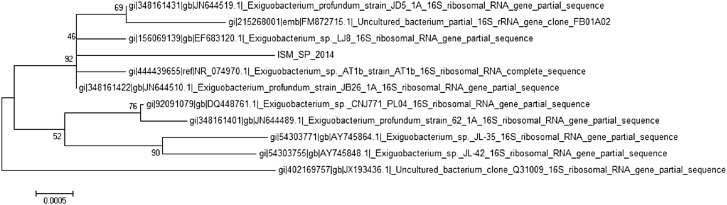
Phylogenetic tree showing relationships between *Exiguobacterium* sp. (ISM SP 2014) and other *Exiguobacterium* sp. (bootstrap = 1000)

In the present work, 44 (9.2%) samples were found contaminated with *E. coli* O157:H7, *L. monocytogenes, Salmonella* sp. and *Exiguobacterium* sp. *E. coli* O157:H7 was found in 0.6% of the RSV. None of the cabbage and coriander samples were found to harbour *E. coli* O157:H7 and *L. monocytogenes*. Only beet-root and spinach show positive results for *E. coli* O157:H7. *Exiguobacterium* sp. (ISM SP 2014) was detected in the spinach sample while studying the bacterial contamination. The 16S rRNA gene sequencing showed less than 92% similarity with sequences available in the public domain (Fig. 2). The presence of *Exiguobacterium* sp. was reported for first time on the surface of RSV.

In this study, *Salmonella* sp. was isolated in 19 (4%) out of 480 samples (7 of spinach, 3 of tomato, 3 of cucumber, 2 each of carrot and beet-root and 1 each of radish and coriander). However, no cases of salmonellosis linked to consumption of salad vegetables were reported in Dhanbad. In a previous study, Kumar (2012) found that 7.8% of salad vegetables harboured *Salmonella* sp. Similarly, Singh et al. (2006) reported the prevalence of *salmonella* spp. in vegetables in northern cities of India with 93.3% of samples containing less than 2.0 log cfu/g, and 16% samples 2.0–7.0 log cfu/g; the study also reported that pond water used for irrigation and cleansing of vegetables and water used by vegetable vendors for sprinkling on vegetables for keeping them fresh might be the most important and primary source of contamination of vegetables with *Salmonella* sp. Similarly, in Middle East, *Salmonella* spp. was detected in 6.7% (*n* = 90) of the raw vegetables from the post-harvest, washing areas of Bekaa Valley, Lebanon and Beirut (Faour-Klingbeil et al. 2016).

In general, occurrence of *L. monocytogenes* on the RSV analysed in this study was comparatively low (2.9%), in accordance with previous results presented by Porto and Eiroa (2001). They reported 3.2% of samples were positive for *L. monocytogenes*. In Canada, Denis et al. (2016) reported that the *L. monocytogenes* was detected in 14 samples out of 4435 of leafy vegetables collected from a wide range of retail stores. However, conflicting results were reported by Ponniah et al. (2010) in a study in Malaysia, where *L. Monocytogenes* was detected in 22.5% of minimally processed vegetables on analysis. *L. monocytogenes* was detected in 14% (*n* = 90) of vegetable samples, collected from Bekaa Valley, Lebanon and Beirut of Middle East (Faour-Klingbeil et al. 2016). Till date, there are no reported cases of listeriosis linked to consumption of foods in the study area; however, *L. monocytogenes* is recognized as an important foodborne pathogen worldwide. In the present work, *Salmonella* sp. and *L. monocytogenes* were the most prevalent species amongst the detected pathogens.

## Conclusions

The present study showed the potential hazard of RSV collected from retail market in Dhanbad city of India. Detection of *Exiguobacterium* sp. from the surface of RSV pointing the adoption of good agricultural practices and good hygienic practices is required to minimize the contamination of RSV. However, the presence of pathogenic bacteria such as *E. coli* O157:H7, *L. monocytogenes* and *Salmonella* spp. in RSV should not be underestimated, particularly for raw consumption. The most possible causes of of vegetable acting as vehicles of the pathogens might be washing of the vegetable with contaminated water or growing the vegetables using contaminated soil or irrigation with contaminated water. Results indicated the need of adoption of hygienic practices by food vendors, processors and consumers to minimize the risks of transmission of pathogens. The results can be useful for identifying microorganisms associated with particular vegetables. In addition, the government must have a better surveillance on the activities of vegetable vendors to minimize the risk of disease outbreak associated with consumption of contaminated vegetables.
